# Serum Osteopontin as a Potential Marker for Metastasis and Prognosis in Primary Osteogenic Sarcoma: A Systematic Review

**DOI:** 10.7759/cureus.60544

**Published:** 2024-05-18

**Authors:** Alok C Agrawal, Dikshant Saini, Rachita Nanda

**Affiliations:** 1 Orthopedics, All India Institute of Medical Sciences, Raipur, Raipur, IND; 2 Orthopedic Surgery, All India Institute of Medical Sciences, Raipur, Raipur, IND; 3 Biochemistry, All India Institute of Medical Sciences, Raipur, Raipur, IND

**Keywords:** osteoblast, therapeutic outcome, serum osteopontin level, osteopontin (opn), osteosarcoma (os), primary osteogenic sarcoma

## Abstract

Osteosarcoma (OS), a primary malignant bone tumor, poses significant challenges in diagnosis and prognosis. It is a painful medical burden, and treating it is still a difficult issue. Osteopontin (OPN), a multifunctional extracellular matrix protein, has emerged as a promising biomarker in this context. This systematic review explores the role of OPN as a diagnostic and prognostic marker in OS, highlighting its potential in enhancing early detection, monitoring disease progression, and predicting patient outcomes. Various studies have demonstrated elevated levels of OPN in OS patients, correlating with tumor aggressiveness, metastatic potential, and poor prognosis. In addition, OPN's involvement in tumor microenvironment regulation and metastatic processes underscores its clinical relevance as a biomarker. For this systematic review, comprehensive literature searches were conducted in the PubMed databases for research published between the database's establishment and November 11, 2022. Out of the nine studies that were available for analysis, a higher level of OPN in primary osteogenic sarcoma patients indicates a poorer prognosis and higher incidence of metastasis. OS has not shown commensurable progress with concerns to treatment approches and survical outcomes. However, the discovery of a biological marker that can predict metastasis and severity will be a groundbreaking development for advancements in OS diagnosis and treatment. Therefore, understanding the intricate interplay between OPN and OS pathogenesis holds promise for improving patient management and developing targeted therapeutic strategies.

## Introduction and background

Osteosarcoma

**Figure 1:**
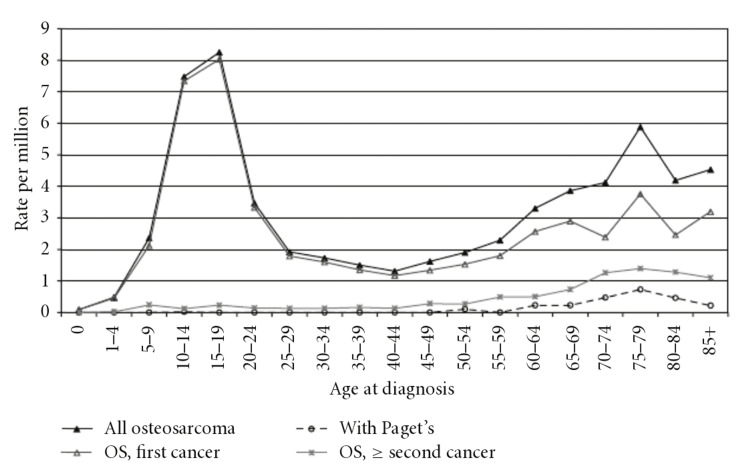
Incidence of osteosarcoma per million population Abbreviations: Os: osteosarcoma Citation: Savage SA, Mirabello L: Using epidemiology and genomics to understand osteosarcoma etiology. Sarcoma. 2011, 2011:548151 [4]

The most common bone cancer in children and adolescents is osteosarcoma (OS) [1]. The incidence rates of OS are four and five cases per million people per year, respectively, for ages 0-14 and 0-19. In India, where this cancer is associated with higher deaths, the incidence ranges from 4.7% to 11.6% [2]. Males are more likely than females to develop OS than vice versa (5.4 vs. 4.0 cases per million individuals per year, respectively). The incidence of OS has two peaks, the first of which occurs between the ages of 10 and 14, during a period of rapid growth, showing a strong correlation between adolescence and OS. Above 65 years, the second peak occurs often secondary to Paget’s disease. The long bones, including the proximal tibia and distal femur, are where the majority of OS originate. OS is extremely aggressive and has a 20% metastatic rate, with the lungs being the typical site of metastasis [3]. Figure 1 depicts data derived from the Surveillance, Epidemiology, and End Results (SEER) program on the population, previously published by Savage and Mirabello [4].

The mainstay treatment is surgery with the excision of the primary lesion alongside administering adjuvant and neoadjuvant chemotherapy. Surgical procedure includes amputation or salvage. Despite the standardized chemotherapy regime, an onset of earlier metastasis can lead to the failure of treatment efficacy subsequently causing death. A significantly better prognosis is seen in patients with primary tumor alone as compared to patients with metastasis. In patients with metastases at baseline, the five-year survival rate was 27.4% and 70% for patients with no metastases [5].

Osteopontin

Osteopontin (OPN) is a chemokine-like calcified extracellular matrix-associated protein first identified in the bone [6]. The 314 amino acid residue human OPN is a highly negatively charged protein that appears to have no complexity in its secondary structure [7]. It has serine-valine-valine-tyrosine-glycine-leucine-arginine and arginine-glycine-aspartate domains for human OPN integrin binding, calcium binding sites, and heparin-binding domains for extracellular matrix receptor III (CD44 antigen-mediated) binding [8]. The Arg-Gly-Asp (RGD) sequence interacts with various integrin receptors, activating regulatory and structural proteins like Src and focal adhesion kinase, initiating diverse signaling pathways involved in biological processes, such as bone remodeling, immune responses, and disease progression [9]. OPN has five isoforms generated through alternative splicing of the secretory phosphoprotein 1 gene, undergoing various post-translational modifications. It is expressed in multiple cell types, including those involved in bone metabolism (osteoblasts and osteoclasts), immune responses (lymphocytes), and certain cancers [9]. OPN-a, OPN-b, and OPN-c are the splice variants that are cancer-specific. Blood levels of certain isoforms of OPN like OPN-b and -c serve as biomarkers for specific malignancies [9].

OPN has emerged as a significant player in the pathogenesis of OS, a primary malignant bone tumor. Its role in OS stems from extensive research exploring the intricate interactions between tumor cells and their microenvironment. Within the historical context, studies have elucidated OPN's multifaceted functions, including regulation of cell adhesion, migration, invasion, and modulation of the immune response.

**Figure 2:**
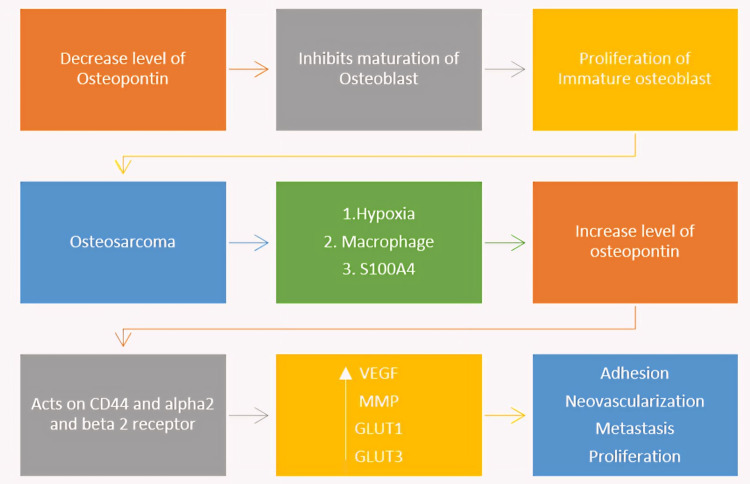
Flowchart depicting OPN relation with OS Abbreviations: S100A4: S100 calcium-binding protein A4; CD44: extracellular matrix receptor III; VEGF: vascular endothelial growth factor; MMP: matrix metalloproteinases; GLUT 1/3: glucose transporter 1/3; OPN: osteopontin; OS: osteosarcoma

OPN is a glycoprotein that helps in the maturation of osteoblasts with the help of Runt-related transcription factor 2 converting the mesenchymal stem cells to mature osteoblast. Due to the dysregulation of the expression of tumor suppressor genes triggered by genetic or epigenetic events or mutation creates a microenvironment for the OS by decreasing the OPN level, thereby preventing the maturation of the osteoblast, as shown in Figure 2. This leads to the proliferation of immature cells. However, as the tumor grows, it needs neovascularization to prevent the hypoxic condition causing the release of the hypoxic inducing factor, which increases the level of OPN and promotes neovascularization.

**Figure 3:**
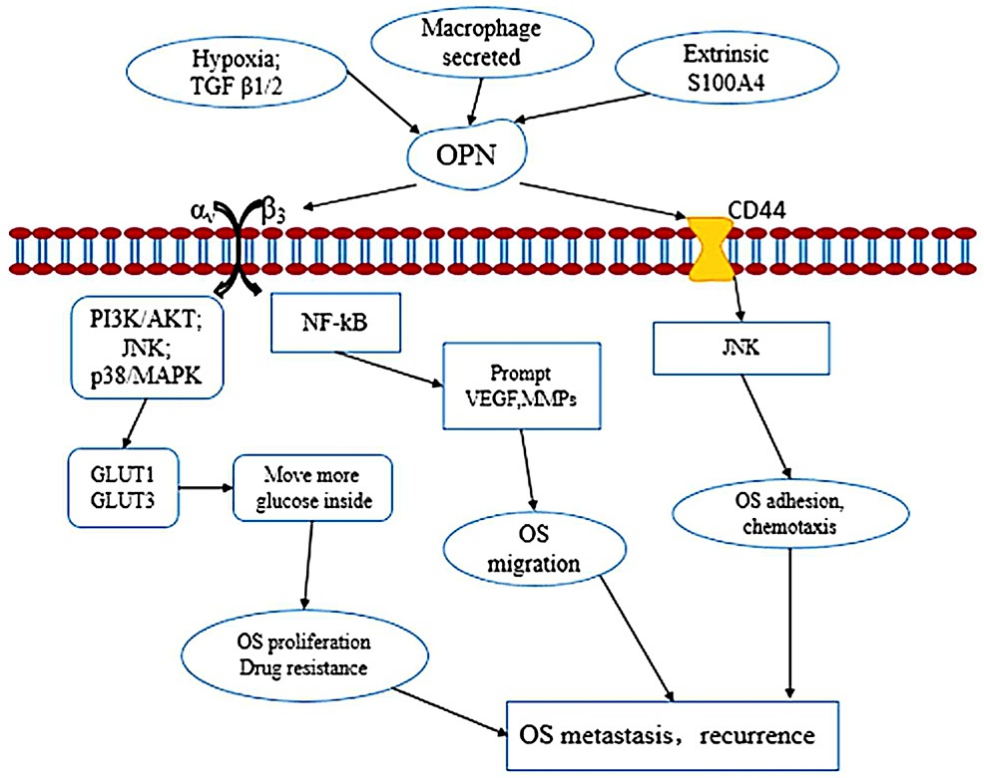
Pathogenesis of osteosarcoma recurrence and metastasis Abbreviations: OS: osteosarcoma; OPN: osteopontin; S100A4: calcium-binding protein A4; TGFβ1/2: transforming growth factor β1/2; VEGF: vascular endothelial growth factor; GLUT1/3: glucose transporters 1/3; MMP: matrix metalloproteinases; PI3K: phosphoinositide-3-kinase; CD44: extracellular matrix receptor III; AKT: protein kinase B; p38: mitogen-activated protein kinase; JNK: c-JUN N-terminal kinase; NF-κB: nuclear factor-κB Citation: Han X, Wang W, He J, Jiang L, Li X: Osteopontin as a biomarker for osteosarcoma therapy and prognosis. Oncol Lett. 2019, 17:2592-8 [9]

Hence, the elevated level of OPN and subsequent neovascularization stimulates the cascade of events leading to increase in GLUT 1 and GLUT 3, causing more uptake of glucose by tumor cells and prolonging their survival and enhancing their proliferation, as described in Figure 3.

However, the excellent efficacy of OPN as a tumor marker is not limited to OS only. Numerous studies have shown a connection between increased OPN secretion and many other cancers, including glioblastoma, breast and prostate cancer, squamous cell carcinoma, melanoma, and OS. In lung cancer, overexpression of OPN is linked to both patient survival and the results of therapeutic interventions like surgery, chemotherapy, or radiotherapy. OPN is a predictor of malignancy and poor results following neoadjuvant chemotherapy in breast cancer [8], and higher OPN levels are linked to a worse prognosis. In gastrointestinal cancer, a higher OPN level is linked to lymph node metastases, the tumor-node-metastases stage, depth of invasion, tumor size, and distant metastasis [9]. In genitourinary tumors, OPN can be utilized as a marker for malignancy and medication resistance.

Osteogenic sarcoma is a painful medical burden, and treating it is still a difficult issue. While an early diagnosis and better treatments have increased the five-year survival of other tumors, the clinical outcomes for OS have not demonstrated commensurable progress. Therefore, there is an urgent need for advancements in OS diagnosis and treatment. The discovery of biological markers that can aid in the diagnosis of OS and can predict metastasis and severity will be a ground-breaking development. The investigation and development of therapeutic approaches for OS patients may be aided by the identification of these specific tumour biomarkers. The historical trajectory of OPN as a biomarker in OS has been marked by a series of studies elucidating its expression patterns in tumor tissues and biological fluids, such as serum. These investigations have highlighted the correlation between elevated OPN levels and tumor aggressiveness, metastatic potential, and patient prognosis. Moreover, the advent of advanced molecular techniques has facilitated the exploration of OPN's molecular mechanisms and its interaction with various signaling pathways implicated in OS pathogenesis. Collectively, these findings underscore OPN's promising utility as a biomarker for early detection, risk stratification, and therapeutic monitoring in OS patients, offering insights into personalized treatment strategies and improved clinical outcomes.

Therefore, we aimed to systematically review studies investigating the role of OPN in OS.

## Review

Objectives

This study aimed 1) to assess the serum OPN level as a marker for severity in primary osteogenic sarcoma, 2) to assess the serum OPN level as a marker for metastasis in primary osteogenic sarcoma, and 3) to assess the serum OPN level as a marker for therapeutic outcome in primary osteogenic sarcoma.

Search strategy

This study was conducted in accordance with the PRISMA guidelines (Preferred Reporting Section for Systematic Review and Meta-analysis). We used the search terms (“osteogenic sarcoma” OR “bone sarcoma” OR “primary osteosarcoma” OR “BONE”) AND (“Osteopontin” OR “Sialoprotein 1” OR “secretory phosphoprotein 1”) OR (“Uropontin” OR “SPP 1” OR “OPN”). Retrieved studies are limited to English only. 

Eligibility criteria

We identified the latest literature on the topic and all the studies showing a relationship with OPN and OS were included. Articles comprising human participants of all genders were included. Articles with free full text online were selected. Other articles and abstracts for whom the full text could not be retrieved were excluded. Animal studies were excluded.

Sources

On November 5, 2022, we searched the PubMed database for OPN and OS. We also performed a snowball search to identify additional studies by searching the reference list for publications eligible for full-text review and using PubMed to identify and filter the respective research citing those references.

Selection process

Two researchers individually reviewed the titles and abstracts of the articles and determined contrariety until a consensus was reached. In the event of disagreement, the third researcher's consensus was taken on which articles should be screened as full-text. Again, if there is disagreement, consensus on inclusion or exclusion has been reached through discussion. All articles went through the eligibility criteria and were shortlisted.

Results

**Figure 4:**
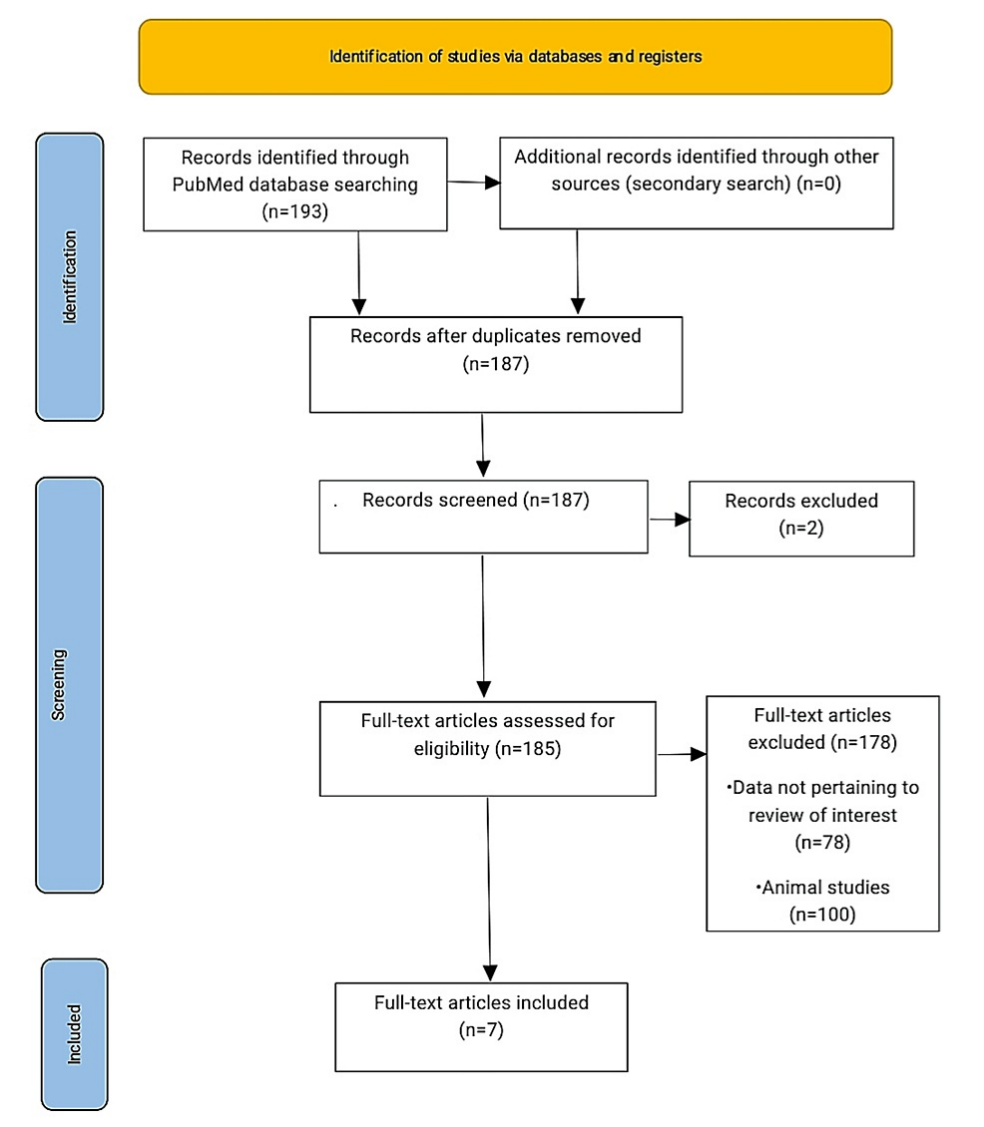
PRISMA flow diagram for the study PRISMA: Preferred Reporting Items for Systematic Reviews and Meta-Analysis

All the shortlisted articles passed through the quality assessment tools. All co-authors did many quality checks. Out of 187 studies from the PubMed database, 100 were animal studies, which were excluded, and 78 did not match the objective. Nine studies were considered for full review, out of which two studies were abstracts. Subsequently, we retrieved the full-text versions of these articles and conducted a thorough quality assessment. Ultimately, we included seven articles in our review. The detailed screening process with the study selection process is represented in Figure 4 within the PRISMA flowchart.

The list of the studies included in this review article is given in Table 1.

**Table 1:** List of studies selected for the review Abbreviations: Runx2: Runt-related transcription factor-2; miR-4262: micro RNA gene; MMP-2: matrix metalloproteinase-2; MMP-9: matrix metalloproteinase-9

	Authors [Reference]	Year of study	Country	Journal	Title of the study
1	Wong et al. [10]	2000	Hong Kong	Clinical Cancer Research	Quantitative Analysis of Circulating Tumor Cells in Peripheral Blood of Osteosarcoma Patients Using Osteoblast-Specific Messenger RNA Markers
2	Nazarizadeh et al. [11]	2021	Iran	Journal of Bone Oncology	Evaluation of Local and Circulating Osteopontin in Malignant and Benign Primary Bone Tumours
3	Liang et al. [12]	2021	China	International Journal of Clinical Oncology	The Cancer-Related Transcription Factor Runx2 Combined With Osteopontin: A Novel Prognostic Biomarker in Resected Osteosarcoma
4	Villanueva et al. [13]	2019	Chile	Journal of Cellular Physiology	The Cancer-Related Transcription Factor RUNX2 Modulates Expression and Secretion of the Matricellular Protein Osteopontin in Osteosarcoma Cells to Promote Adhesion to Endothelial Pulmonary Cells and Lung Metastasis
5	Hsieh et al. [14]	2014	Taiwan	PLOS ONE	Osteopontin Upregulates the Expression of Glucose Transporters
6	Song et al. [15]	2016	China	Tumour Biology	Regulation of Osteosarcoma Cell Invasion Through Osteopontin Modification by miR-4262
7	Liu et al. [16]	2004	China	Chinese Medical Journal	Effect of Human Osteopontin on Proliferation, Transmigration and Expression of MMP-2 and MMP-9 in Osteosarcoma Cells

An overview of the studies selected for this review is summarized in Table 2. 

**Table 2:** Baseline characteristics of the samples of the selected studies. Abbreviations: OS: osteosarcoma; RUNX-2: Runt-related transcription factor 2; SaOS: sarcoma osteogenic; MG63: human osteoblastic cell line; U2OS: human bone osteosarcoma epithelial cell; HOS: human osteosarcoma cell; G292: human osteosarcoma cell; 143B: cell line derivative of the HOS cell line; miR-4262: microRNA-4262; MMP-2/9: matrix metalloproteinases-2/9; RPMI 1640: Roswell Park Memorial Institute 1640

Authors	Study name	Sample size	OS patients	Healthy individuals
Wong et al. [10]	Quantitative Analysis of Circulating Tumor Cells in Peripheral Blood of Osteosarcoma Patients Using Osteoblast-Specific Messenger RNA Markers: A Pilot Study	40	11	29
Nazarizadeh et al. [11]	Evaluation of Local and Circulating Osteopontin in Malignant and Benign Primary Bone Tumours	182	27	29
Liang et al. [12]	The Cancer-Related Transcription Factor Runx2 Combined With Osteopontin: A Novel Prognostic Biomarker in Resected Osteosarcoma	105	105	0
Authors	Study name	Osteosarcoma cell lines		
Villanueva et al. [13]	The Cancer-Related Transcription Factor RUNX2 Modulates Expression and Secretion of the Matricellular Protein Osteopontin in Osteosarcoma Cells to Promote Adhesion to Endothelial Pulmonary Cells and Lung Metastasis	SaOS‐2, MG63, U2OS, HOS, G292, and 143B		
Hsieh et al. [14]	Osteopontin Upregulates the Expression of Glucose Transporters in Osteosarcoma Cells	MG63, U-2OS, and 143B		
Song et al. [15]	Regulation of Osteosarcoma Cell Invasion Through Osteopontin Modification by miR-4262	U2OS		
Liu et al. [16]	Effect of Human Osteopontin on Proliferation, Transmigration and Expression of Mmp-2 and Mmp-9 in Osteosarcoma Cells	OS cell cultured in vitro using RPMI 1640 cell culture medium vector: *E. coli* BL21 (DE3) cells		

The summary of the results of the selected studies is given in Table 3.

**Table 3:** Summary of results of the selected studies Abbreviations: OS: osteosarcoma; OPN: osteopontin; RUNX-2: Runt-related transcription factor-2; sOPN: serum osteopontin; CoCl2: cobalt((II) chloride; GLUT 1/3: glucose transporter 1/3; MG63: human osteoblastic cell line; U-2OS: human bone osteosarcoma epithelial cell; 143B: cell line derivative of HOS cell line; shOPN 1/2: human osteopontin plasmid 1/2; MMP 2/9: matrix metalloproteinases 2/9; SaOS-2: sarcoma osteogenic cell line 2; MG63: human osteoblastic cell line; U2OS: human bone osteosarcoma epithelial cell; HOS: human osteosarcoma cell; G292: human osteosarcoma cell; IgG: immunoglobulin G; miR-4262: microRNA gene

Authors	Study name	Results
Wong et al. [10]	Quantitative Analysis of Circulating Tumor Cells in Peripheral Blood of Osteosarcoma Patients Using Osteoblast-Specific Messenger RNA Markers: A Pilot Study	The mRNA levels of osteocalcin, osteonectin, OPN, and type I collagen in peripheral blood were elevated in 91% of OS patients, but were elevated only in 35% of healthy people [10].
Nazarizadeh et al. [11]	Evaluation of Local and Circulating Osteopontin in Malignant and Benign Primary Bone Tumours	Among a cohort of 29 healthy individuals, the serum osteopontin levels ranged from 10.06 to 13.35 ng/ml [11]. Within a group of 153 patients diagnosed with bone tumors, 72 individuals with benign bone tumors exhibited serum osteopontin levels ranging from 46.68 to 65.68 ng/ml, while 27 osteosarcoma patients demonstrated higher serum osteopontin levels, ranging from 218.92 to 308.41 ng/ml [11].
Hsieh et al. [14]	Osteopontin Upregulates the Expression of Glucose Transporters in Osteosarcoma Cells	Treatment with 100 µM of CoCl2 significantly increased OPN levels in OS cell lines, with a threefold induction observed after six hours and a 1.6-fold induction after 12 hours [14]. Exogenous administration of 10 ng/ml of OPN led to a dose-dependent upregulation of GLUT1 and GLUT3 expression in U2O(S) and 143B cell lines, with GLUT1 levels increasing by 2.25-fold in U2O(S) OS cell line and 1.75-fold in 143B OS cell line, and GLUT3 levels increasing by twofold in both cell lines after 24 hours [14]. In MG 63 osteosarcoma (OS) cell lines, infection with an empty vector resulted in a significant increase in GLUT1 and GLUT3 expression levels, reaching threefold compared to the control. However, upon transinfection with shOPN1 and shOPN2, GLUT1 levels were reduced to 1.5 times and two times the control, respectively, while GLUT3 levels decreased to two times and 1.75 times the control, respectively [14].
Liang et al. [12]	The Cancer-Related Transcription Factor Runx2 Combined With Osteopontin: A Novel Prognostic Biomarker in Resected Osteosarcoma	The study consisting of 105 osteosarcoma patients demonstrates a positive correlation between the expression levels of the cancer-related transcription factor Runx2 and osteopontin (OPN) in 55 patients and a negative correlation in 50 patients [12]. This suggests that as the expression of Runx2 increases, so does the expression of OPN, indicating a potential regulatory relationship between these two biomarkers in osteosarcoma.
Liu et al. [16]	Effect of Human Osteopontin on Proliferation, Transmigration and Expression of Mmp-2 and Mmp-9 in Osteosarcoma Cells	In osteosarcoma cell lines with metastasis, serum OPN levels were significantly elevated (>64 ng/ml) compared to normal cell lines (14-64 ng/ml) [16]. Cell cycle analysis revealed that the presence of OPN was associated with an increase in the proportion of cells in the S phase (4.8% vs. 3% when OPN was absent). Additionally, a higher percentage of cells were found in the G0/G1 phase in the presence of OPN (14.11 +/- 3.88%) compared to when OPN was absent (9.29 +/- 2.08%) [16]. These results suggest a potential role for OPN in promoting cell cycle progression and metastatic behavior in osteosarcoma.
Villanueva et al. [13]	The Cancer-Related Transcription Factor RUNX2 Modulates Expression and Secretion of the Matricellular Protein Osteopontin in Osteosarcoma Cells to Promote Adhesion to Endothelial Pulmonary Cells and Lung Metastasis	Preincubation with antibody lowers cell adhesion by 25-50% when compared to control tests without antibody or with IgG control. RUNX2 regulates OPN production and helps osteosarcoma cells adhere to pulmonary endothelial cells. RUNX2 and OPN are associated with osteosarcoma metastasis [13].
Song et al. [15]	Regulation of Osteosarcoma Cell Invasion Through Osteopontin Modification by miR-4262	It was observed that there was a notable decrease in the level of miR-4262 and a significant increase in levels of OPN in osteosarcoma specimens in contrast to corresponding adjacent non-tumor tissue.

Discussion 

Serum OPN as a Marker for Prognosis in OS

Of the studies included in this review, three of them primarily focused on evaluating the role of OPN in OS. These studies concluded that OPN could be employed as an independent biomarker for determining therapeutic efficacy and prognosis in OS patients and highlighted the association of OPN with micrometastases in patients with OS.

The pilot study conducted by Wong et al. consisting of 11 OS patients and 29 healthy individuals showed that in 91% of OS patients, OPN levels were high, but on the other hand, in only 35% of normal healthy individuals, it was raised. It was also found that six OS patients with relatively higher serum OPN levels developed clinical metastasis within 12 months of diagnosis. Further studies have shown that OPN can be used as a biomarker for metastasis and prognosis for various cancers along with OS as in the case of multiple tumors consisting of ovarian, gastric, lung, breast, and melanoma [9]. They have described the functional and structural characteristics of OPN along with concluding the unique role played by OPN in the proliferation and metastasis of malignant cells. The interaction of OPN with hypoxia-inducible factor and the promotion of E-cadherin to help tumor metastasis is also shown [9]. OPN helps in the maturation of osteoblasts while OS involves the proliferation of immature cells. This indicates that, initially, the OPN level should be low for the proliferation of tumor cells, but for the cells to metastasize, the OPN level should be high. However, to obtain a more standard reference value for OPN, proper case-control studies with diverse population strata need to be carried out.

The study by Nazarizadeh et al. evaluated local and circulating OPN in benign primary and malignant bone tumors. This study demonstrates a clear disparity in serum OPN levels between OS patients and healthy individuals, with metastatic tumor patients exhibiting the highest levels. Serum OPN level was assessed in 153 patients classified into two groups: patients diagnosed with benign primary bone tumors (n = 72) and patients with malignant primary bone tumors (n = 81) [11]. A control group comprising healthy individuals (n = 29) was additionally included. Out of the 81 patients with malignant bone tumors, 27 were OS patients. Results showed that serum OPN levels were the highest in malignant primary bone tumors, followed by benign primary bone tumors, and were the lowest among the control group. It highlighted that OPN levels could predict metastasis, grade, and prognosis. It also showed that OPN mRNA expression in patients with OS receiving chemotherapy was 1.5 times lower than the untreated counterparts. The OPN level was 1.7 times higher in OS patients with metastasis in comparison to OS patients with no metastasis. Moreover, the OPN level was two times lower in OS patients with no recurrence in comparison to patients having a recurrence. The only limitation of this study was that it focused on OPN as a marker for primary bone tumor, but it was not specific for OS, and the survival analysis was not carried out for a satisfactory duration.

The study by Liang et al. aimed to explore the prognostic significance of the role played by the Runt-related transcription factor 2/OPN axis in the metastasis of OS cells. The study included a total of 105 clinical samples from OS patients without prior treatment and substantiated the expression of OPN and Runt-related transcription factor 2 through immunohistochemistry. This study also showed a positive correlation between the expression levels of the cancer-related transcription factor Runx2 and OPN in 55 patients and a negative correlation in 50 patients. Out of the 55 patients with positive OPN correlation, 34 patients developed lung metastasis, a tumor size of <8 cm was found in 42 of these patients, and 27 patients showed good histological response. Meanwhile, out of the 50 patients with negative OPN correlation, only nine patients developed lung metastasis, a tumor size of >8 cm was seen in 13 patients, and 16 patients showed good histological response. Results suggested the possibility of a combined expression of Runx2/OPN as a valuable independent predictor of metastasis and overall survival in OS [12]. Combined consideration of OPN and Runx2 showed extensions in predictive range and an improvement in sensitivity, leading to a probable novel prognostic biomarker. Although the study aims to emphasize the combined role of RunX2 and OPN as a prognostic tool in resected OS, it sheds limited theoretical explanation about tumor metastasis and survival. The results lack to establish the consistency of future trends of outcomes obtained, which could be substantiated with a proper patient follow-up taken over a time frame of six months.

Serum OPN as a Marker for Severity in OS

The study by Hsieh et al. investigates how in OS, OPN enhances the expression of glucose transporters. In tumor proliferation and survival, glucose acts as a crucial source of metabolic energy. Typically, many types of tumors showcase an overexpression of OPN and glucose transporters like GLUT1 and GLUT3 (hypoxia-responsive). It is associated with tumorigenesis and metastasis. For the study, the human OS cell lines MG63, U-2OS, and 143B were taken under prime consideration [14]. Control shRNA was used as a negative control. CoCl2 was used to mimic the hypoxic condition as an inevitable cellular stress experienced during tumor progression and solid tumor development which upregulated OPN mRNA and protein expression in OS cells. It was also demonstrated that GLUTs can be upregulated by both CoCl2 and OPN. Hence, the evaluation of glucose uptake indicated that OPN increases nutrient availability to OS cells. Subsequently, the knockdown of OPN expression was performed in the OS cell lines MG63 and U-2OS using two different shRNA plasmids, which decreased the cell survival by approximately 20% in OPN knockdown cells. Hypoxia-induced expression of GLUTs was also inhibited by OPN knockdown. As a result, it was observed that both glucose transporters and OPN were upregulated in hypoxic human OS cells. Another glucose transporter inhibitor, i.e., phloretin, also caused cell death by treatment alone. Comparatively, the phloretin-induced cell death was significantly enhanced in OPN knockdown OS cells to 80%. Synergistic cytotoxic effects were exhibited in three OS cell lines by a combination of a low dose of phloretin and chemotherapeutic drugs, such as daunomycin, 5-Fu, etoposide, and methotrexate. Inhibition of glucose transporters markedly potentiated the apoptotic sensitivity of chemotherapeutic drugs in OS [14]. However, a cellular-based study might be short in result consistency and sensitivity with control and non-control group patients on a larger sample size. Moreover, the study is based on the GLUT-OPN relationship and provides a potential chemotherapeutic treatment for OS and only indirectly provides the OPN role in prognosis with respect to other simultaneous factors involved in OS patients.

The study conducted by Liu et al. explored in vitro the mechanism in which OPN affects matrix metalloproteinases (MMP) 2 and 9 (MMP-2, MMP-9) to proliferate, transmigrate, and express in OS cells. MMPs have significant roles in the growth of tumor cells, migration, invasion, metastasis, and angiogenesis. The study was carried out by the production of a prokaryotic expression vector of human OPN and subcloning it into *E. coli* BL21(DE3) cells [16]. Furthermore, the proliferation, cell cycle, and expression of cyclin A in OS cells were investigated by using MTT assay, flow cytometry, and Western blot, respectively [16]. A transwell cell culture chamber was used to check the transmigration of OS cells. The chemotaxis of hOPN to OS cells was studied by a micro-pore-filter-membrane system. The levels of total protein were examined according to Coomassie Brilliant Blue manuals followed by evaluation of the expression of MMP-2 and MMP-9 by detecting the volume of degradation of gelatin on SDS-PAGE gel. The resultant prokaryotic-expression vector of hOPN and purified hOPN protein were achieved, indicating that hOPN promoted OS cell proliferation in a dose-dependent manner along with stimulating cyclin A expression in OS cells to accelerate cell division cycle. hOPN has chemotaxis to OS, which increases the transmembrane migration of OS cells and also enhances the secretion of MMP-2 and MMP-9 in OS cells [16]. The study establishes the correlation between MMP and hOPN in a summarized manner but does not provide an extensive investigation to assess the consistency of outcomes. OS cells in the presence of OPN were more in the S phase of the cell cycle as compared to the less number in the G0/G1 cycle, which helped in the multiplication of this cell. However, this study was an in vitro study. No factual explanation about the direct prognostic role of MMP positive expression with respect to OPN in OS has been made. The study is exclusively in vitro; hence, in order to strengthen the findings and further test the clinical applications of determining the influence of MMP-2, MMP-9, and hOPN on the metastasis of OS patients, well-designed clinical trials with bigger human sample sizes must be carried out. However, these results suggest a potential role for OPN in promoting cell cycle progression and metastatic behavior in OS.

Serum OPN as a Marker for Metastasis in OS

One of the studies conducted by Villanueva et al. intended to investigate the combined expression of Runt-related transcription factor 2 (RUNX-2) and OPN in OS patients as an independent and significant prognostic marker and predictor of OS metastasis and survival. The study by Villanueva et al. shows that OPN receptors (ITGB1, ITGB3, ITGB5, ITGA4, ITGA5, ITGA6, ITGA7, ITGA8, ITGA9, and ITGAV) and CD44 and CXCR were expressed on both OPN and pulmonary endothelial cells, therefore suggesting that OPN secretion helps OS cells to adhere with pulmonary endothelial cells. OPN expression is directly controlled by RUNX2 [13]. The study primarily focused on RUNX2 rather than OPN. It mainly focused on treatments that were targeted toward reducing pulmonary metastasis in OS. However, it gave an indirect clue that elevated OPN via RUNX2 mediated pulmonary cell adhesion and caused pulmonary metastasis. It was a cell line-based study with no proper case and controls being taken. Even though the study talks about lung metastasis and treatment to prevent metastasis, it does not comment on metastasis elsewhere. It also does not deal with the relationship of OPN with the severity and prognosis of OS. However, the study strongly establishes that RUNX2 and OPN are associated with OS metastasis and hence the probable efficacy of OPN as a biomarker.

The study by Song et al. demonstrated OS specimens had significantly decreased levels of miR-4262 and increased levels of OPN as compared to the paired adjacent non-tumor tissue, which helped in cell invasion. The patients with high miR-4262 levels had better five-year survival than the patients with lower miR-4262 levels in the resected OS. Furthermore, bioinformatics analyses showed that miR-4262 targeted the 3'-UTR of OPN mRNA to inhibit its translation, which was proved by a luciferase reporter assay. It was also seen that miR-4262 overexpression inhibited OPN-mediated cell invasion, while miR-4262 depletion increased OPN-mediated cell invasion in OS cells, in both a transwell cell invasion assay and a scratch wound healing assay [15]. The compilation study data suggested that silencing of miR-4262 in OS cells promotes OPN-mediated invasion [15], while also elucidating the prognostic significance of OPN modification by miRNAs in OS patients. However, a further detailed study to substantiate the findings might be of more clinical and therapeutic significance.

## Conclusions

Primary osteogenic sarcoma is a tumor of immature osteoblasts, whereas OPN helps in the maturation of osteoblasts; henceforth, their relationship is quite controvertible. OPN expression is essential for the maturation of osteoblasts via integrin avß3-mediated cell signaling, and OPN is essential for controlling osteoblast development. In comparison to differentiated mature osteoblasts, OS cells exhibit much lower levels of OPN expression. Reduced OPN levels impede mesenchymal stem cells' (MSCs') ability to differentiate into OBs, which is consistent with the finding that a tumor is a proliferation of cells that are not fully differentiated. Decreased OPN expression inhibits the maturation of mesenchymal stem cells or immature osteoblasts into mature osteoblasts but maintains the features of immature osteoblastic-like cells, which may lead to OS. Altered levels of serum OPN concentrations may be linked to aberrant differentiation, proliferation, and maturation of OS cells. Reduced OPN in OS cells implies that most OS cells do not undergo terminal osteogenic differentiation which promotes OS. Nonetheless, it has been noted that higher OPN levels in stromal or tumor cells increase OS's capacity to spread. In tumors, glucose transporters are overexpressed and associated with metastasis. OPN increases glucose uptake through integrin αvβ3 and promotes the hypoxia-dependent expression of GLUT-1 and GLUT-3 (glucose transporters).

Another interesting finding is that under hypoxic conditions, OSs exhibit an upregulation of OPN due to increased hypoxia-inducing factors. Furthermore, it has been observed that dysregulated expression of microRNA (miRNA) contributes to the growth and spread of cancer. Overexpression of miR-4262 inhibits OPN-mediated invasion, while reduced miR-4262 promotes invasion of OS cells. In addition, upon the growth of a tumor size larger than 1 cm and the onset of hypoxia within the tumor cells, there is an increase in the expression of VEGF (vascular endothelial growth factor), which is dependent on HIF1α. This occurs through the stimulation of the p65 subunit of NF-κB (nuclear factor-κB) by integrin-linked protein kinase B, which in turn promotes tumor angiogenesis. Upregulated expression of tumor cells derivative OPN increases metastatic property of tumor cells. Although clinical studies have highlighted higher levels of OPN expression in OS patients, COX-2 expression, and association with VEGF, serum OPN is not predictive of outcomes in OS patients. The results of the above studies indicate that elevated expression of OPN induces a cascade of events leading to several changes in the tumor metastasis mechanism. This implies that by monitoring OPN levels, clinicians can better assess the prognosis of OS patients and tailor treatment strategies accordingly, ultimately improving patient care and outcomes.

Further research with clinical studies is needed to confirm the role of serum OPN to substantiate the diagnostic efficiency in OS, which may provide insights about tumor pathogenesis, distinguishing defects and metastasis in OS. Sufficient blood samples should be collected from OS patients and healthy controls to conduct clinical trials and analyze OPN expression in normal blood samples to obtain a standard serum OPN reference value for diagnosing OS, predicting metastasis in OS, and concluding prognosis. Future studies may provide answers to the following questions: i) if the increased level of serum OPN is due to elevated circulating OS cells, and ii) if the increased level of serum OPN correlates with OS grade, circulating OS cell count, disease-free survival and metastasis. However, the studies reviewed in this article suggest that OPN has emerged as a significant player in the pathogenesis of OS. Collectively, the findings of these studies emphasize OPN's promising utility as a biomarker for early detection, risk stratification, and therapeutic monitoring in OS patients, offering insights into personalized treatment strategies and improved clinical outcomes.
